# Enhancement of the efficacy of cancer chemotherapy by the pineal hormone melatonin and its relation with the psychospiritual status of cancer patients

**Published:** 2010

**Authors:** Giuseppina Messina, Paolo Lissoni, Paolo Marchiori, Erio Bartolacelli, Fernando Brivio, Luciano Magotti

**Affiliations:** a Psychological Service, Fondazione IRCCS, Ospedale Maggiore Policlinico, Mangiagalli e Regina Elena, Milan, Italy; b Institute of Biological Medicine, Milan, Italy; c Natur Spiritual, Milan, Italy; d Psychological Service, Bassini Hospital, Cinisello Balsamo, Milan, Italy; e Surgery Division, Bassini Hospital, Cinisello, Milan, Italy; f Foundation Legrenzi-Cazzullo, Milan, Italy

**Keywords:** Bioimmunomodulation, Chemotherapy, Lung Cancer, Melatonin, Psychoncology, Spirituality

## Abstract

**BACKGROUND:**

The anti-oxidant and immunomodulating natural agents may enhance the efficacy of cancer chemotherapy. One of the most important agents is the pineal hormone melatonin (MLT) which may exert both anti-oxidant and antiproliferative immunostimulating anticancer effects. This study was performed to evaluate the efficacy of a biochemotherapeutic regimen in metastatic cancer patients, and its therapeutic activity in relation to the psychospiritual status of patients.

**METHODS:**

The study included 50 metastatic non-small cell lung cancer (NSCLC) patients and a control group of 100 patients. Chemotherapy consisted of cisplatin plus gemcitabine. MLT was given orally at 20 mg/day in the evening. Patients were subdivided into 5 psychic profiles, as follows: spiritual faith, rationale faith, anxiety, apathy, and accusation behavior.

**RESULTS:**

Tumor response rate was significantly higher in patients treated by chemotherapy plus MLT than in those treated by chemotherapy alone (21/50 vs. 24/100, p < 0.001). However, the percentage of objective tumor regressions obtained in patients with spiritual faith was significantly higher than that found in the overall other patients concomitantly treated by chemotherapy plus MLT (6/8 vs. 15/42, p < 0.01).

**CONCLUSIONS:**

In conclusion, the efficacy of chemotherapy may be enhanced by the pineal hormone MLT, by representing a new promising biochemotherapeutic combination; also despite its objective ability to enhance chemotherapy efficacy, the activity of MLT is depending at least in part on the psychospiritual status of cancer patients, and it is maximal in the presence of a real spiritual faith.

The possibility to amplify the anticancer efficacy of chemotherapy by a bioimmunomodulatory approach represents one of the most promising future therapeutic strategies of cancer.^1^,^2^ At present, the most commonly used chemobiotherapeutic regimen is consisting of the association between chemotherapy and monoclonal antibodies against growth factor receptors or angiogenic substances,^3^,^4^ in an attempt to control cancer cell proliferation and angiogenic processes. However, before the clinical use of monoclonal antibodies in the treatment of human neoplasms, clinical and experimental studies had already demonstrated the possibility to enhance the cytotoxic potency of cancer chemotherapy by anti-oxidant agents.^5^ One of the most potent anti-oxidant agents available in the nature is represented by the pineal hormone melatonin (MLT).^6^ MLT would act as an anticancer agent through its neuroendocrine action on specific cell receptors rather than as a simple antioxidant drug, and through its antiproliferative and immunomodulating effects,^7^–^9^ and in particular it has been shown that the concomitant administration of pharmacological doses of MLT may increase the therapeutic efficacy of cancer chemotherapy, particularly in patients with poor clinical status.^10^ Several other natural anticancer agents, such as Aloe extracts, and their association with cancer chemotherapy seem to deserve prosing results in the treatment of human neoplasms.^11^ On the other hand, some preliminary clinical studies have suggested that the efficacy of cancer chemotherapy may also depend on the psychospiritual status of cancer patients,^12^ particularly in non-small cell lung cancer (NSCLC). This finding would be due to a different functional status of the psychoneuroendocrinoimmune (PNEI) system, which is the biological structure mediating the influence of emotions and consciousness status on the clinical history of the neoplastic disease, namely by regulating the anticancer immune response.^13^–^15^ The possibility to enhance the efficacy of cancer chemotherapy through a biological neuroimmune modulation is at present a scientific evidence, and the biological agent most commonly used to pilot host immunobiological response in an antitumor way and enhance the efficacy of cancer chemotherapy is the pineal hormone MLT.^6^–^10^ However, at present no study has been carried out to evaluate whether the psychospiritual status may influence not only chemotherapy alone, but also its association with biological agents, such as MLT. Therefore, the present study was performed to investigate the possible influence of the psycospiritual status of cancer patients on a chemobiotherapeutic regimen with classical chemotherapy in association with effective anticancer immunomodulatory agents, such as the pineal hormone MLT.

## Methods

The study included 50 consecutive metastatic NSCLC patients who were treated by chemotherapy consisting of cisplatin (CDDP) plus gemcitabine (GEM) in association with the pineal hormone MLT. The clinical protocol was explained to each patient, and informed consent was obtained. CDDP was given iv at a dose of 30 mg/m^2^ /day for 3 consecutive days, and GEM was injected iv at 1000 mg/m^2^ at days 1 and 8, corresponding to one complete chemotherapeutic cycle. Cycles were repeated every 21 day-intervals for at least 3 cycles. MLT was given orally at a pharmacological dosage of 20 mg/day in the evening, in accordance with its physiological light/dark circadian rhythm^6^ every day without interruption and starting 7 days prior to the onset of chemotherapy. The clinical response was assessed according to WHO criteria, and patients were evaluated radiologically after 3 cycles of chemotherapy. The clinical characteristics of patients are shown in table 1.

**Table 1:** Clinical characteristics of 50 patients suffering from metastatic NSCLC

Characteristics	n
Male/Female	35/15
Median performance status (Karnofsky’s score)	90 (8-100)
Histotypes:	
Epidermal carcinoma	17
Adenocarcinoma	27
Large cell carcinoma	6
Dominant metastasis sites:	
Nodes	5
Bone	7
Lung	27
Liver	8
Lung + Liver	3

The results were compared to those observed in a control group of 100 histotype- and disease extension-matched patients treated by the same chemotherapy alone. Moreover, as far as the psychospiritual evaluation is concerned, according to the results of a psychological analysis consisting of a specific patient questionnaire^12^ and a psychological interview to evaluate the emotional reaction to the diagnosis of disease and their acceptance of the treatment, patients were subdivided into five main psychic profiles as follows: spiritual faith, rationale faith, anxiety, apathy and accusation behavior. The spiritual faith was defined not only on the basis of the presence of a religious believe, but also on that of spiritual sensitivity and lack of anxiety, self-punishment and unconscious projections against other people. On the contrary, as previously published,^16^ the rationale faith was defined as lack of any emotional involvement in the reaction against the disease, without any real spiritual involvement. Anxiety was assessed by a specific psychological score. Apathy was consisting of a complete indifference toward the disease and treatment itself. Finally, accusation behavior was defined as the presence of projections against people and world as a possible cause of the cancer. Data were analyzed using the student’s t test.

## Results

An objective tumor regression, consisting of complete response (CR) or partial response (PR), was achieved in 21/50 (42%) patients treated by chemotherapy plus MLT, but in only 24/100 (24%) control patients treated by chemotherapy alone. This difference was statistically significant (p < 0.001). However, as reported in table 2, considering the efficacy of chemotherapy plus MLT in relation to the psychospiritual status of patients, the best results were observed in patients with evidence of spiritual faith, and these results were significantly different compared to the overall other patients who received chemotherapy plus MLT (6/8 (75%) vs. 15/42 (36%), p < 0.01). In particular, the percentage of tumor regressions achieved in sceptic and misanthropic patients concomitantly treated by MLT, even though higher, was not significantly different from that in patients treated with the only chemotherapy. The percentage of disease control (DC), consisting of CR plus PR plus stable disease (SD), was also significantly higher in patients with spiritual faith than in the other chemobiologically treated patients (p < 0.05).

**Table 2:** Clinical response (WHO criteria) obtained in 50 metastatic non-small cell lung cancer patients treated by chemotherapy (cisplatin plus gemcitabine) and the pineal hormone melatonin in relation to their psychospiritual status.

Psychospiritual status	Clinical response +						
	n	CR	PR	CR + PR	SD	DC	PD
Spiritual faith	8	2	4	6 (75%) *	1	7 (88%) **	1
Rationale faith	15	1	6	7 (47%)	2	9 (60%)	6
Anxiety	12	0	4	4 (33%)	3	7 (58%)	5
Apathy	10	0	3	3 (30%)	1	4 (50%)	6
Accusation behavior	5	0	1	1 (20%)	1	2 (40%)	3

* p < 0.01 vs. the other groups

** p < 0.05 vs. the other groups

+ CR: complete response; PR: partial response; SD: stable disease; DC (CR + PR + SD): disease control

## Discussion

According to previous data,^6^–^10^ these results confirm that the pineal hormone MLT may enhance the efficacy of cancer chemotherapy, at least in NSCLC patients. Moreover, this study shows that the chemical action of MLT, despite its potential capacity of improving the efficacy of cancer chemotherapy, does not abrogate the importance of the psychospiritual status of cancer patients in influencing the prognosis of their neoplastic disease. In fact, the best results with MLT have been obtained in psychospiritual patients. On the contrary, the concomitant administration of MLT did not induce clinically evident therapeutic effects in patients who had no spiritual sensitivity. In more detail, the presence of psychological characteristics, such as scepticism and misanthropy, seems to reduce the efficacy of not only chemotherapy, but also its possible biomodulation by natural anticancer biological agents, such as the pineal hormone MLT. These results further confirm the capacity of MLT to enhance the anticancer efficacy of chemotherapy, at least in lung cancer.

## Conclusions

Despite the real potential therapeutic anticancer activity of the biological natural agents, such as MLT itself, it does neither abrogate, nor magnify the importance of the psychospiritual status of patients in relation to the occur rence of their neoplastic disease. However, the too small sample size does not allow drawing definitive conclusions. So, further randomized clinical studies are required to confirm these promising results.
